# Comment on Mitamura et al. Treatment Strategies for Locoregional Recurrence in Esophageal Squamous-Cell Carcinoma: An Updated Review. *Cancers* 2024, *16*, 2539

**DOI:** 10.3390/cancers17203333

**Published:** 2025-10-16

**Authors:** Keiichi Jingu

**Affiliations:** Department of Radiation Oncology, Tohoku University Graduate School of Medicine, 1-1 Seiryo-chou, Aoba-ku, Sendai 980-8574, Japan; keiichi.jingu.e8@tohoku.ac.jp; Tel.: +81-22-717-7312; Fax: +81-22-717-7316

I read with great interest the article entitled “Treatment Strategies for Locoregional Recurrence in Esophageal Squamous-Cell Carcinoma: An Updated Review” by Mitamura et al. [1] However, I would like to point out the following three points.

1. Omitted prospective evidence

First, I noted that the authors did not cite two prospective studies on postoperative recurrence of esophageal cancer, which are also the only two prospective reports across all treatment modalities, including surgery, radiotherapy, and chemotherapy [2,3]. In these studies, the median overall survival (OS) durations were 27 months (*n* = 48, 95% confidence interval [CI] 5.278–49.58 months) and 21 months (*n* = 30, 95% CI 2.5–39.5 months). Mitamura et al. [1] reported that the surgical outcomes were superior; however, their conclusion was based on the analysis of retrospective data collected from select patients in whom surgery was feasible and whose general condition allowed surgical intervention. Although it was a small cohort and retrospective study, Jingu et al. also reported the results of chemoradiotherapy for isolated lymph node metastases after curative surgery, and showed that the median OS of patients with supraclavicular lymph node metastases who were suitable for surgery was particularly good, at 117.5 months (*n* = 6, 95% CI not available) [4].

2. Immunotherapy outcomes

Second, I believe that the review’s implication regarding the efficacy of immunotherapy combined with chemotherapy requires cautious interpretation. In the CheckMate 648 trial [5], cited in the review, the prognosis was better with immunotherapy combined with chemotherapy than chemotherapy alone in patients with unresectable advanced or recurrent esophageal squamous cell carcinoma. However, in patients with locoregional recurrence, the median OS durations were 14.8, 13.9, and 13.5 months in the nivolumab plus chemotherapy, nivolumab plus ipilimumab, and chemotherapy alone groups, respectively, revealing a lack of significant improvement. This crucial finding was not clearly presented in the review, potentially leading to data misinterpretation.

3. Importance of local treatment approaches

Finally, Wu et al. retrospectively demonstrated that the median progression-free survival of patients with locoregional recurrence was 11.27 (*n* = 11, 95% CI 2.45–20.09) months and 4.17 (*n* = 28, 95% CI 2.64–5.71) months in the radiotherapy and non-radiotherapy group, respectively (*p* = 0.081). The median OS duration was 19.48 (*n* = 11, 95% CI 8.37–30.60) months in patients receiving immunotherapy with radiotherapy, which was significantly longer than that observed in those receiving immunotherapy without radiotherapy (*n* = 28, 7.69 months, 95% CI 3.45–11.93 months; *p* = 0.026) [6]. Therefore, in the absence of a specific reason, such as the inability to perform radiotherapy, immunotherapy alone may not be recommended as monotherapy for postoperative locoregional recurrent esophageal cancer.

In light of these findings, I believe that the suggested algorithm of treatment strategies for postoperative locoregional recurrent esophageal squamous cell carcinoma, as presented in the review by Mitamura et al., has limitations. Moreover, the algorithm allows for the omission of essential local treatment approaches, such as surgery and radiotherapy.
